# Regulation of T cell differentiation and function by long noncoding RNAs in homeostasis and cancer

**DOI:** 10.3389/fimmu.2023.1181499

**Published:** 2023-06-06

**Authors:** Julia Erber, Dietmar Herndler-Brandstetter

**Affiliations:** Center for Cancer Research and Comprehensive Cancer Center, Medical University of Vienna, Vienna, Austria

**Keywords:** noncoding RNA, tumor-infiltrating T cell, regulatory T cell, gamma delta T cell, exhaustion, dysfunction, tumor immune evasion, adoptive cell therapy

## Abstract

Long noncoding RNAs (lncRNAs) increase in genomes of complex organisms and represent the largest group of RNA genes transcribed in mammalian cells. Previously considered only transcriptional noise, lncRNAs comprise a heterogeneous class of transcripts that are emerging as critical regulators of T cell-mediated immunity. Here we summarize the lncRNA expression landscape of different T cell subsets and highlight recent advances in the role of lncRNAs in regulating T cell differentiation, function and exhaustion during homeostasis and cancer. We discuss the different molecular mechanisms of lncRNAs and highlight lncRNAs that can serve as novel targets to modulate T cell function or to improve the response to cancer immunotherapies by modulating the immunosuppressive tumor microenvironment.

## 1 Introduction

Estimates suggest that less than 3% of transcripts in the human genome are protein-coding (1). According to large-scale projects such as ENCODE, at least 80% of the mammalian genome is actively transcribed, and the majority is noncoding RNAs (ncRNAs) (1–3). NcRNAs are molecules that lack a protein-coding sequence. Within the noncoding transcriptome, small noncoding RNAs (sncRNAs) (< 50 bp in size) (4, 5) can be distinguished from long noncoding RNAs (lncRNAs) (> 200 bp in size) (6). Different sncRNAs have been characterized such as microRNAs (miRNAs), transfer RNAs (tRNAs), PIWI-interacting RNAs (piRNAs), small nuclear RNAs (snRNAs) and small interfering RNAs (siRNAs) (4, 5). Interestingly, ncRNAs can function as therapeutic targets, and the first RNAi drug has been approved for clinical use in 2018 (7, 8). Although numerous differentially regulated lncRNAs in immune cells and cancer have been described (2, 3, 6), no lncRNA-targeting therapeutics are in clinical use yet (8).

All lncRNAs except circular RNAs (circRNAs) are classified based on their relative position to the loci of protein-coding genes and enhancers: (1) long intergenic noncoding RNAs (lincRNAs), (2) (anti-)sense lncRNAs, (3) intronic lncRNAs, (4) bidirectional RNAs and (5) enhancer RNAs (eRNA) (9–11). Most lncRNAs are capped by 7-methyl guanosine (m^7^G), spliced less efficiently than mRNAs, and are polyadenylated and transcribed by RNA polymerase II. Though, eRNAs are mostly bidirectionally capped transcripts that are not spliced or polyadenylated and that are primarily unstable (12). And circRNAs are produced by back-splicing where an exon is spliced to an upstream instead of a downstream exon (13).

The question of how many lncRNAs are located in the human genome is challenging to answer as there are considerable discrepancies between automated annotations and the highly-curated ENCODE collection (10, 14). The number of lncRNAs reported in recent human genome annotations ranges from 27,919 to 58,648. Due to functional and transcriptional noise, the exact number of functional lncRNAs is still debated. Yet, the cellular function of a growing number of lncRNAs has been identified experimentally (15, 16).

Interestingly, the number of lncRNAs is associated with the complexity of an organism, and species- and cell type-specific expression is frequently observed (17, 18). Although the majority of lncRNAs are not evolutionarily conserved, there are still thousands of syntonic (conservation at genomic position) and sequence-based conserved lncRNAs (17, 19). Yet, in a study by Guo and colleagues, several conserved lncRNAs are processed differently in human embryonic stem cells (ESC) compared to murine ESC, resulting in different subcellular location and function (20). These findings indicate that the mode of lncRNA conservation across species is not yet completely understood.

Even though lncRNAs are expressed on average at a lower level compared to protein-coding genes, which generate more RNA molecules per transcriptional burst (21), lncRNAs engage through modular domains with DNA, RNA or proteins (12). Thereby, lncRNAs have crucial functions in many different cellular processes, including chromatin regulation (22, 23), genome integrity (24, 25), regulation of transcription in *cis* and *trans* (26), nuclear organization (27) and post-transcriptional functions (28). Even though thousands of lncRNAs have been identified, only a limited number of lncRNAs have been studied to determine their biological significance, such as the role of Xist in X chromosome silencing (29) and H19 in genomic imprinting (30).

T lymphocytes are a heterogeneous group of immune cells that include cytotoxic CD8^+^ T cells, CD4^+^ helper T (Th) cells, regulatory T cells (Tregs), gamma delta (γδ) T cells and natural killer T cells (NKT cells). The development, differentiation and function of these T cell subsets is tightly regulated by transcriptional programs and epigenetic processes (31–33). Naïve CD4^+^ T cells can differentiate into various effector cell lineages, such as Th1, Th2 and Th17 cells, which secrete different effector molecules and thereby characteristically shape the tumor microenvironment (TME) and the prognosis of cancer patients (34–36). Cytotoxic CD8^+^ T cells mediate tumor cell killing by recognizing tumor neoantigens and enhanced CD8^+^ T cell infiltration into tumors, in particular those with a high tumor mutational burden (TMB), is associated with better prognosis in almost all solid cancers (36). However, an immunosuppressive TME and chronic stimulation by tumor antigens can lead to dysfunctional CD8^+^ T cells, referred to as exhausted CD8^+^ T cells (CD8^+^ Tex) (37, 38). In addition, the absence of interleukin (IL)-21-expressing CD4^+^ T cells or increased levels of IL-10 and transforming growth factor beta (TGF-β) in the TME have been implicated in the formation and persistence of exhausted CD8^+^ T cells (CD8^+^ Tex) (39, 40). CD8^+^ Tex cells are incapable of effectively utilizing their effector functions even in early stages of malignancy (41, 42). CD8^+^ Tex cells express inhibitory receptors, such as PD-1, LAG3, CXCR5, TIM-3, CD244 (also known as 2B4 or SLAMF4), CD38, CD39 and CD101 (43–48) as well as transcription factors, such as Tox, TCF-1, T-bet and Eomes (38, 40, 49–53). So far, two major subtypes of CD8^+^ Tex cells have been described: early PD-1^int^ (progenitor or stem-like) and late PD-1^hi^ (terminally) exhausted T cells (44, 53). PD-1^int^ early Tex cells can be defined by the expression of TCF-1 and T-bet (40, 52, 53), while PD-1^hi^ Tim-3^+^ late Tex express Tox, Eomes and regulator of G protein signaling 16 (Rgs16) (38, 40, 49–51, 54, 55). A recent study even suggests a 4-stage CD8^+^ Tex cell model, which is regulated by the TCF-1/T-bet/Tox axis and comprises quiescent, resident Tex^prog1^ (PD-1^+^ Ly108^+^ CD69^+^), Tex^prog2^ (PD-1^+^ Ly108^+^ CD69^–^), Tex^int^ (PD-1^+^ Ly108^–^ CD69^–^) and terminally exhausted, resident Tex^term^ cells (PD-1^+^ Ly108^–^ CD69^+^) (54). The role of lncRNAs in CD8^+^ T cell exhaustion and its impact on tumor immune evasion and the effectiveness of T cell-targeted therapies, such as immune checkpoint blockade or adoptive T cell therapy, will be discussed in section “2.1 CD8^+^ T cells”.

Here we highlight recent advances in the role of lncRNAs in regulating the differentiation and function of T cell subsets in homeostasis and cancer. We review which lncRNAs can serve as promising targets to modulate T cell function or to improve the response to cancer immunotherapies by modulating the immunosuppressive tumor microenvironment. We conclude by highlighting the challenges that still lie ahead for the translation of research findings to the clinics and the development of effective lncRNA-based anti-cancer therapies.

## 2 The role of lncRNAs in T cells during homeostasis and cancer

**Figure 1:**
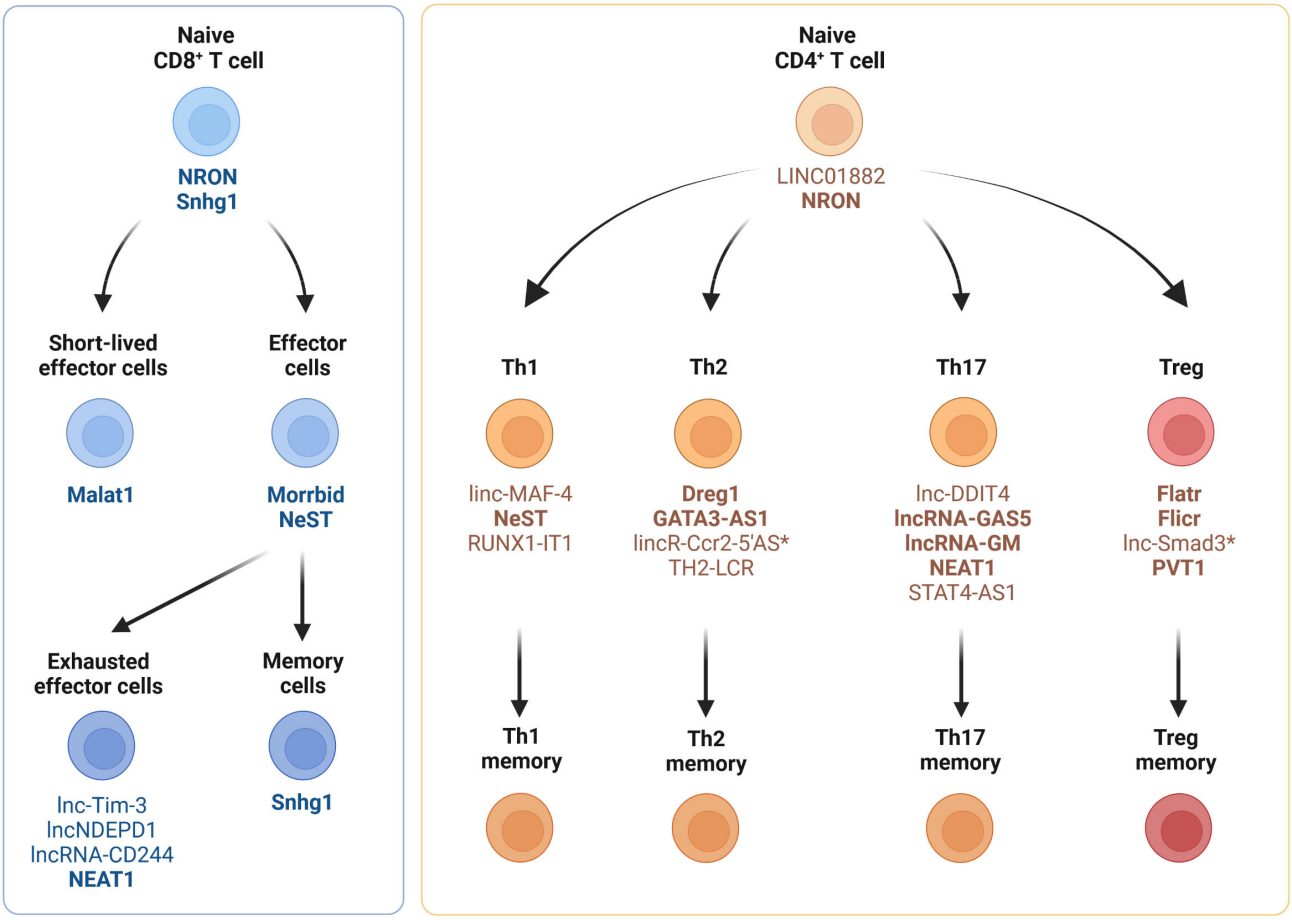
Expression of lncRNAs in CD4^+^ and CD8^+^ T cell subsets. Cell type-specific and differentiation-specific lncRNAs that modulate CD4^+^ or CD8^+^ T cell function or differentiation during homeostasis, infection and autoimmunity are shown. Th1, CD4^+^ T helper 1 cells; Th2, CD4^+^ T helper 2 cells; Th17, CD4^+^ T helper 17 cells; Treg, regulatory T cells. LncRNAs expressed in humans and mice (bold), lncRNAs expressed solely in mice (*).

In this chapter and its associated four subchapters, we provide an overview of the expression of lncRNAs in T cell subsets (CD8^+^ T cells, CD4^+^ T cells, regulatory T cells, γδ T cells and NKT cells) during homeostasis and cancer. A large number of lncRNAs has been shown to be expressed in immune cells, including mouse and human T cell subsets (56–61). Of these studies, two demonstrated that CD4^+^ and CD8^+^ T cells can be distinguished based on their lncRNA expression profile (56, 57). Spurlock et al. performed whole-genome sequencing to identify CD4^+^ T cell lineage-specific lncRNAs. They reported that lncRNAs are selectively expressed in human T cells differentiated under Th1-, Th2- and Th17-polarizing conditions (56). These findings are consistent with another study by Hudson et al., demonstrating in humans and mice, that naïve, effector and memory CD8^+^ T cell populations display unique lncRNA expression profiles (57). They also demonstrate that lncRNAs with synteny or sequence conservation across the two species show a comparable lncRNA expression landscape during differentiation. This implies that lncRNAs may regulate cell states and cell fate decisions during T cell activation and differentiation (56, 57, 62). **Figure 1** provides an overview of the expression of lncRNAs in naïve, effector and memory CD8^+^ and CD4^+^ T cell subsets in mice and humans.

Thousands of lncRNAs have been shown to be expressed in cancer cells, metastases and tumor stroma (5, 63–65). Intriguingly, many of the genomic mutations in cancer cells reside inside regions that are transcribed into lncRNAs (66, 67), and the expression and dysregulation of lncRNAs are strikingly cancer-type specific (68). In Chapter 4, we will therefore highlight tumor cell-expressed lncRNAs that drive immune escape by modulating T cell infiltration or T cell dysfunction/exhaustion, and that may therefore represent promising targets for therapeutic intervention.

However, only limited information is available on the expression and role of lncRNAs in tumor-infiltrating T cell subsets and how they affect T cell function, tumor immune escape and the efficacy of T cell-based cancer immunotherapies. Recently, the first comprehensive catalog and functional collection of lncRNAs in human tumor-infiltrating T cells was generated by performing single-cell RNA-sequencing (scRNA-seq) of T cell libraries for three cancer types (62). In this study, 154 lncRNA signature genes were associated with effector, exhausted and regulatory T cell stages of which 84 lncRNAs may modulate T cell function according to functional annotation. Even though the expression profiles and mechanisms were not addressed, this study provides the foundation for future studies of lncRNAs regulating effector and exhausted tumor-infiltrating T cells. In the following four subchapters, we will highlight the most important lncRNAs expressed in T cell subsets (CD8^+^ T cells, CD4^+^ T cells, regulatory T cells, γδ T cells and NKT cells), describe their mechanism and summarize their relevance during homeostasis and cancer.

### 2.1 CD8^+^ T cells

Although many lncRNAs have been shown to be differentially expressed between CD8^+^ T cell subsets in mice and humans, only a few lncRNAs have been experimentally validated for their biological relevance to CD8^+^ T cell differentiation and function (**Figure 1** and **Table 1**). Resting naïve CD8^+^ and CD4^+^ T cells express lncRNA NRON (noncoding repressor of NFAT), which limits excessive T cell activation (79–81) and nuclear factor of activated T cells (NFAT)-dependent cytokine production (81). Effector CD8^+^ T cell differentiation and function are regulated by several other lncRNAs, such as Malat1, NeST and Morrbid (**Figure 1**). The chromatin-associated lncRNA Malat1 promotes differentiation of short-lived effector cells (SLECs), thereby impairing the generation of memory precursor effector cells (MPEC) and long-lived memory cells (74). Mechanistically, Malat1 directly interacts with EZH2, the catalytic subunit of the polycomb repressive complex 2 (PRC2), thus leading to increased H3K27me3 deposition at memory cell-associated genes. The lncRNA NeST (also known as IFNG-AS1 or Tmevpg1) controls microbial susceptibility by epigenetic activation of the IFN-γ locus in CD8^+^ T cells (77) and regulates IFN-γ in Th1 cells (78). NeST RNA binds to WDR5, a component of the histone H3-lysine 4 (H3K4) methyltransferase complex, thus altering histone 3 methylation at the IFN-γ locus. The lncRNA Morrbid and its locus have been shown to control CD8^+^ T cell expansion, survival and effector function during viral infection by regulating the pro-apoptotic factor *Bcl2l11* (also known as Bim) and PI3K-AKT signaling strength (84). However, Morrbid is not only expressed in CD8^+^ T cells but also regulates the survival of neutrophils, eosinophils and classical monocytes via transcriptional regulation of *Bcl2l11* (84). Finally, lncRNA SNHG1 has been shown to promote memory CD8^+^ T cell differentiation via IL-7-mediated signaling as well as initiation of a transcriptional memory differentiation program via the STAT3-TCF1-Blimp1 axis (85).

**Table 1:** LncRNAs regulating CD8^+^ T cells, γδ T cells and NKT cells during homeostasis and cancer.

LncRNA	LncRNA type	Molecular Interaction	Cancer type	T cell subset	Species	Affected Signaling Molecules/Pathway	Function	Ref.
**Exosome lncRNAs**	–	–	HCC	CD8^+^ T cells	h	–	Promotion of exhaustion phenotype in non-exhausted CD8^+^ T cells within the TME via exosome-associated lncRNAs secreted from exhausted CD8^+^ T cells.	(69)
**LIN00240**	intergenic	lncRNA- RNA	CC	NKT cells	h	miR-123-3p/ STAT3/MICA	NKT cell tolerance and cervical cancer progression is greatly promoted by LINC00240 regulation of STAT3 and MICA.	(70)
**lncNDEPD1**	intergenic	lncRNA- RNA	NSCL C	CD8^+^ T cells	h	Notch1/lncNDEPD1/ miR-3619-5p/PD-1	In PD-1^high^ CD8^+^ T cells, Notch1 upregulates lncNDEPD1/miR- 3619-5p and promotes PD-1^high^ expression.	(71)
**LncRNA- CD244**	antisense	lncRNA- protein	–	CD8^+^ T cells	h	CD244/lncRNA- CD244/Ezh2	CD244 signaling upregulates lncRNA-CD244, which represses IFN-γ and TNF-α transcription by recruiting Ezh2 to deposit H3K27me3 to IFN-γ and TNF-α promoters.	(72)
**lnc-Tim-3**	intergenic	lncRNA- protein	HCC	CD8^+^ T cells	h	Tim-3/Lck/NFAT1/AP-1	Lnc-Tim-3 interacts with Tim-3 to release Bat3, suppressing Lck/NFAT1/AP-1 signaling while inducing MDM2 and Bcl2. Thus, lnc-Tim-3 induces CD8^+^ T cell exhaustion in HCC.	(73)
**Malat1**	intergenic	lncRNA- protein	–	CD8^+^ T cells	h, m	Malat1/Ezh2	Interaction of Malat1 with Ezh2 induces H3K27ac chromatin modifications to repress the memory cell transcription program, thereby promoting short-lived effector cells.	(74)
**MIAT**	intergenic	–	HCC	CD8^+^ T cells, Treg cells	h, m	PD-1/CTLA-4	LncRNA MIAT correlates with Tregs and exhausted CD8^+^ T cells expressing the co-inhibitory receptors PD-1 and CTLA-4 in HCC.	(75)
**NEAT1**	intergenic	lncRNA- RNA	HCC	CD8^+^ T cells	h, m	miR-155/Tim-3	LncRNA NEAT1 inhibits miR-155/Tim-3, induces CD8^+^ T cell apoptosis and limits anti-tumor activity against HCC.	(76)
**NeST**	intergenic, antisense	lncRNA- protein	–	CD8^+^ T cells, CD4^+^ Th1	h, m	NeST/WDR5	NeST binds to WDR5, a component of the H3K4 methyltransferase complex, thus increasing H3K4me3 at the IFN- γ locus in CD8^+^ and CD4^+^ Th1 cells.	(77, 78)
**NKILA**	sense	lncRNA- protein	BC, LC	CD8^+^ T cells, CD4^+^ Th1	h	Ca^2+^/ calmodulin/ NFκB/ STAT1	NKILA regulates T cell sensitivity to AICD by inhibiting NFkB activity and its anti-apoptotic target genes via IkBa.	(28)
**NRON**	intronic, sense	lncRNA- protein	–	CD8^+^ T cells, CD4^+^ T cells	h, m	NRON/NFAT-IQGAP- CK1-GSK3-DYRK	NRON (noncoding repressor of NFAT) regulates nuclear trafficking of NFAT via an RNA-protein scaffold complex to limit excessive T cell activation and NFAT-dependent cytokine production.	(79–81)
**Snhg1**	intergenic	lncRNA- protein	–	CD8^+^ T cells	h, m	Snhg1/Vps13D/IL- 7/STAT3/TCF1/Blimp1	LncRNA Snhg1 binds to Vps13D, thereby inducing IL-7Rα membrane localization and IL-7 signaling via STAT3/TCF1/Blimp1 to trigger memory differentiation.	
**SNHG16**	intergenic	lncRNA- RNA	BC	γδ T cells	h	miR-16-5p/ TGF-β/SMAD5	LncRNA SNHG16 is transmitted by breast cancer-derived exosomes and initiates CD73 expression in γδ1 Tregs by sponging miR-16-5p and inducing SMAD5.	(82)
**TANCR**	–	lncRNA- DNA	–	γδ T cells	h	TRAIL	LncRNA TANCR positively modulates TRAIL expression in *cis* in activated γδ T cells.	(83)

AICD, activation-induced cell death; BC, breast cancer; CC, cervical cancer; HCC, hepatocellular carcinoma; LC, lung cancer; NSCLC, non-small cell lung cancer; h, human; m, mouse.

**Figure 2:**
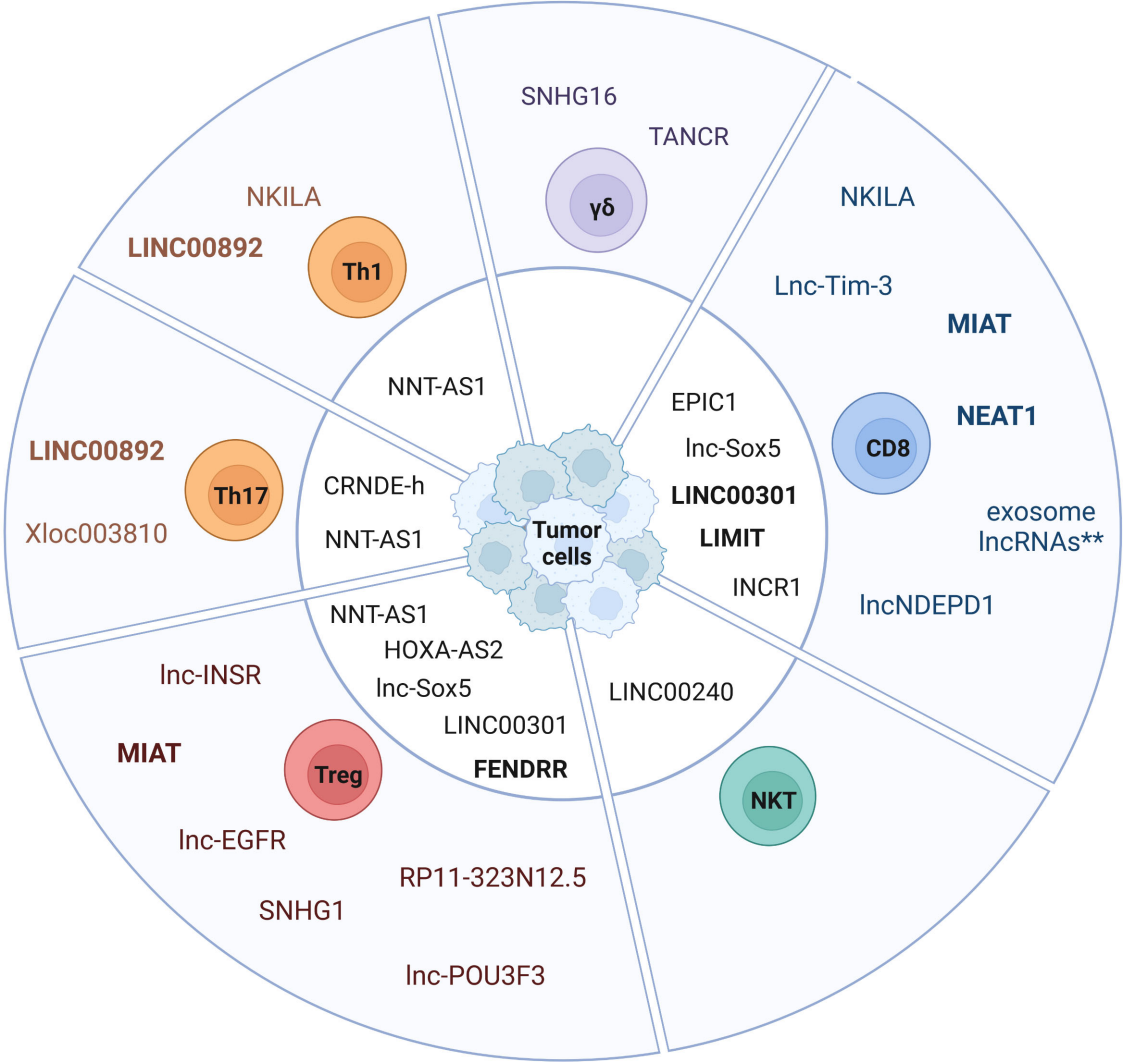
Expression of lncRNAs in tumor-infiltrating T cell subsets. The pie chart depicts lncRNAs expressed in tumor-infiltrating T cell subsets and tumor cell-expressed lncRNAs affecting TIL-specific functions (center). Exosome-derived lncRNAs represent a cluster of lncRNAs that are transmitted from exhausted CD8^+^ T cells via exosomes to non-exhausted CD8^+^ T cells (**) (69). LncRNAs expressed in humans and mice are labeled in bold.

In cancer, cytotoxic CD8^+^ T cells mediate tumor cell killing by recognizing tumor neoantigens and enhanced infiltration of tumors by CD8^+^ T cells is associated with better prognosis in most solid cancers (36). Importantly, TMB and a T cell-inflamed gene expression profile can be used as a pan-cancer biomarker to identify responders and non-responders to anti-PD-1 antibody therapy (86, 87). However, an immunosuppressive TME and chronic stimulation by tumor antigens can lead to dysfunctional CD8^+^ Tex cells. LncRNAs have been shown to play a key role in regulating CD8^+^ T cell function and dysfunction in cancer (**Table 1** and **Figures 1**, **2**). Here we highlight the role and molecular mechanism of lncRNAs associated with tumor-infiltrating CD8^+^ T cells, including lncRNAs that modulate CD8^+^ T cell exhaustion (69, 73, 75) and tumor immune evasion (28, 76, 88). This is of therapeutic significance, since knockdown of lncRNAs lncNDEPD1 and INCR improved the effectiveness of CAR T cell therapy (71, 89).

High expression of PD-1 and co-expression of molecules such as Tim-3 or CD244 on CD8^+^ T cells has been associated with chronic T cell receptor activation and impaired effector function (38, 90). In a recent study, lncNDEPD1 has been shown to regulate PD-1 expression on CD8^+^ T cells in NSCLC patients. Interestingly, knockdown of lncNDEPD1 in chimeric antigen receptor (CAR) T cells reduced tumor growth (71). LncNDEPD1 may therefore represent an interesting target to improve adoptive T cell therapy.

Two lncRNAs that modulate CD8^+^ T cell exhaustion via the co-inhibitory receptor Tim-3 have been identified in hepatocellular carcinoma (HCC) patients (73, 76). Lnc-Tim-3, a NF-κB-modulating lncRNA, was uncovered by high-throughput screening of CD8^+^ T cells originating from TILs of HCC patient samples. Lnc-Tim-3 expression highly correlated with Tim-3^+^ Tex cells and specific binding of the lncRNA to Tim-3 blocked its ligand Bat3 from binding. Consequently, Bat3 facilitated the expression of NF-κB-targeted genes like Bcl-2 and MDM2, thereby promoting survival and exhaustion (73). LncRNA NEAT1 was identified in CD8^+^ T cells from peripheral blood mononuclear cells (PBMCs) of HCC patients. Mechanistically, NEAT1 increases Tim-3 levels by binding miR-155. SiRNA-mediated knockdown of Neat1 limits apoptosis of CD8^+^ T cells and improves tumor cell killing (76).

CD244 is another molecule co-expressed on exhausted PD-1^+^ Tox^+^ CD8^+^ T cells in cancer (38) and chronic viral infection (48). Blockade of CD244 signaling using an anti-CD244 antibody reversed exhaustion in virus-specific CD8^+^ T cells (91). Mechanistically, lncRNA-CD244 is induced by signaling via CD244 in CD8^+^ T cells and represses IFN-γ and TNF transcription by recruiting the N-methyltransferase EZH2 to IFN-γ and TNF promoters to induce H3K27 methylation (72).

**Figure 3:**
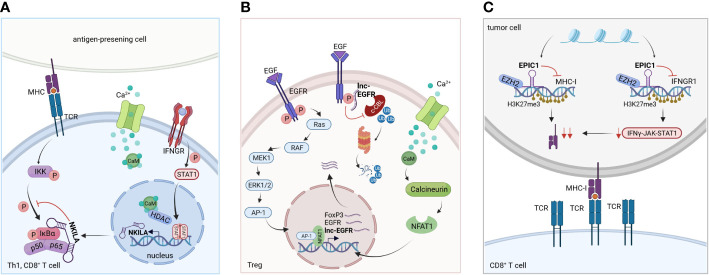
Molecular mechanism of selected lncRNAs. **(A)** LncRNA NKILA: Following T cell activation, IFNγ-JAK-STAT1 signaling triggers NKILA transcription. STAT1-mediated transcription of NKILA is induced by Ca^2+^ influx and activation of calmodulin, which removes HDACs from the promotor. Consequently, NKILA directly interacts with the NFκB-subunit p65 and IκBα, regulating the sensitivity to AICD of Th1 and CD8^+^ T cells. Based on Huang et al. (28). **(B)** LncRNA EPIC1: EPIC1 together with EZH2 enhances H3K27me3 chromatin modifications at the loci of IFNGR1 and antigen presentation genes, downregulating their transcription. This leads to tumor immune evasion and promotes resistance to immune checkpoint inhibitor therapy by blockage of IFNγ-JAK-STAT1 signaling. Based on Guo et al. (92). **(C)** LncRNA lnc-EGFR: EGFR is stabilized by the bound lnc-EGFR, thereby blocking its ubiquitination by c-CBL. EGFR activation and signaling via AP-1/NF-AT1 is enhanced, thereby promoting EGFR expression, Treg cell differentiation and tumor progression. Based on Jiang et al. (93).

In a study by Huang et al., lncRNA NKILA was shown to promote tumor immune escape. Through functional assays in breast and lung cancer, they uncovered that NKILA sensitizes T cells (cytotoxic CD8^+^ T cells and CD4^+^ Th1 cells) to activation-induced cell death (AICD), and high expression of NKILA was associated with reduced patient survival (28). Following T cell activation, IFNγ-JAK-STAT1 signaling induces NKILA transcription. NKILA then binds and inactivates NF-κB (**Figure 3A**), thereby initiating AICD in CD8^+^ T cells and CD4^+^ Th1 cells but not CD4^+^ Tregs and CD4^+^ Th2 cells, which shifts the balance towards an immunosuppressive TME (28). Inhibition of NKILA expression may thus represent a promising target for adoptive T cell therapy and improve therapeutic outcome.

Altogether, these studies showcase how lncRNAs regulate exhaustion, apoptosis and cytotoxicity in tumor-infiltrating CD8^+^ T cells. This could open up possibilities to modify these processes to circumvent tumor immune evasion and increase the effectiveness of T cell-targeted immunotherapies, including adoptive T cell therapies.

### 2.2 CD4^+^ T cells

CD4^+^ helper T cells (Th) are highly versatile and polyfunctional cells that display phenotypic plasticity and heterogeneity depending on the microenvironment. Naïve CD4^+^ T cells can differentiate into various subsets, such as (1) inflammatory Th1 cells, which promote anti-tumor activity through IFN-γ and TNF-α (94), (2) Th2 cells that may contribute to tumorigenesis and tumor progression (95, 96), (3) Th17 cells that have been associated with poor prognosis in hepatocellular carcinoma and thyroid cancer (97), and (4) regulatory T cells that suppress immune responses in the TME (98).

A multitude of lncRNAs has been shown to regulate different CD4^+^ T cell lineages, which is summarized in **Figures 1**, **2** and **Table 2**. Naïve CD4^+^ T cells express lncRNAs LINC01882 (103) and NRON (79). Mechanistically, lncRNA NRON restricts excessive activation in CD4^+^ and CD8^+^ T cells (80, 81). Well-characterized lncRNAs that are expressed in Th1 cells or that modulate Th1 cell differentiation are linc-MAF-4 (105), NeST (77, 117), and RUNX1-IT1 (111). LncRNAs expressed in Th2 cells or that regulate Th2 cell differentiation are Dreg1 (100), GATA3-AS1 (101), lincR-Ccr2-5’AS (104) and TH2-LCR (56). LncRNAs expressed in Th17 cells or that modulate Th17 cell differentiation are lnc-DDIT4 (106), lncRNA-GAS5 (107), lncRNA-GM (108), NEAT1 (109) and STAT4-AS1 (112). LncRNAs that are expressed in Tregs or affect Treg cell differentiation will be discussed in Chapter 2.3.

**Table 2:** LncRNAs regulating CD4^+^ T cell subsets during homeostasis and cancer.

LncRNA	LncRNA type	Molecular Interaction	Cancer type	T cell subset	Species	Affected Signaling Molecules/Pathway	Function	Ref.
**CRNDE-h**	intergenic, antisense	lncRNA- protein	CRC, Glioma, HCC, LC	Th17	h	RORγ/IL-17	CRNDE-h is a tumor exosome-transmitted lncRNA promoting Th17 cell differentiation by inhibiting Itch-mediated ubiquitination and degradation of RORγt in CRC.	(99)
**Dreg1**	antisense	–	–	Th2	h, m	Dreg1/Gata3	Dreg1 induces Gata3 expression in Th2 cells.	(100)
**GATA3- AS1**	antisense	lncRNA- protein	–	Th2	h	WDR5/GATA3/IL-5/ IL-4/IL-13	GATA3-AS1, neighboring the *Gata3* locus, induces Th2 lineage commitment by interacting with the methyltransferase WDR5 and inducing GATA3, IL-5 and IL-13 expression.	(101)
**LINC00892**	intergenic, sense	–	NHL	CD4^+^ T cells	h, m	–	LINC00892 is induced by T cell activation and expressed in CD4^+^ T helper and follicular lymphoma T helper cells.	(102)
**LINC01882**	intergenic, antisense	–	–	CD4^+^ T cells	h	LINC01882/ZEB1/MA P2K4	LINC01882 is expressed in naïve CD4^+^ T cells. Its knockdown increased the expression of transcription factor ZEB1 and MAP2K4, both linked to IL-2 signaling.	(103)
**linc-Ccr2- 5′AS**	intergenic	–	–	Th2	m	Ccr1/Ccr2/Ccr3/Ccr5	Linc-Ccr2-5′AS and the transcription factor GATA3 represent a key regulatory network for Th2-specific cell differentiation and migration.	(104)
**linc-MAF-4**	intergenic	lncRNA- DNA	–	Th1	h	EZH2/LSD1/MAF-4	Linc-MAF-4 modulates Th1 differentiation and function through interaction with EZH2 and LSD1 to epigenetically repress MAF.	(105)
**Lnc-DDIT4**	–	lncRNA- protein	–	Th17	h	Lnc-DDIT4/DDIT4/ mTOR signaling	Lnc-DDIT4 induces DDIT4 expression, which blocks mTOR signaling and inhibits IL-17 production.	(106)
**LncRNA- GAS5**	sense	lncRNA- protein	–	Th17	h, m	GAS5/STAT3	lncRNA-GAS5 inhibits Th17 differentiation by modulating STAT3.	(107)
**lncRNA- GM**	–	lncRNA- protein	–	Th17	h, m	lncRNA-GM/Foxo1	lncRNA-GM directly inhibits Foxo1 dephosphorylation, thus promoting IL-23R expression and Th17 differentiation.	(108)
**NEAT1**	intergenic	lncRNA- protein	–	Th17	h, m	NEAT1/STAT3	NEAT1 reduces STAT3 levels, thereby blocking Th17 cell differentiation in PBMCs of rheumatoid arthritis patients.	(109)
**NeST**	intergenic, antisense	lncRNA- protein	–	CD8^+^ T cells, CD4^+^ Th1	h, m	NeST/WDR5	NeST binds to WDR5, a component of the H3K4 methyltransferase complex, thus increasing H3K4me3 at the IFN- γ locus in CD8^+^ and CD4^+^ Th1 cells.	(77, 78)
**NKILA**	sense	lncRNA- protein	BC, LC	Th1, CD8^+^ T cells	h	Ca^2+^/ calmodulin/ NFκB/STAT1	NKILA regulates T cell sensitivity to AICD by inhibiting NFkB activity and its anti-apoptotic target genes via IkBa.	(28)
**NNT**	intergenic, antisense	–	–	CD4^+^ T cells	h	IFN-γ signaling	NNT is expressed in activated CD4^+^ T cells and is localized near the IFN-γ receptor gene, indicating a functional relationship.	(110)
**NRON**	intronic, sense	lncRNA- protein	–	CD8^+^ T cells, CD4^+^ T cells	h, m	NRON/NFAT-IQGAP- CK1-GSK3-DYRK	NRON (noncoding repressor of NFAT) regulates nuclear trafficking of NFAT via an RNA-protein scaffold complex to limit excessive T cell activation and NFAT-dependent cytokine production.	(79–81)
**RUNX1-IT1**	intronic, antisense	lncRNA- protein	–	Th1	h	RUNX1- IT1/p53/NrCAM	RUNX1-IT1 regulates NrCAM through interacting with p53, thereby modulating T-bet and CXCL10 levels in Graves’ disease.	(111)
**STAT4-AS1**	sense	lncRNA- protein	–	Th17	h	STAT4-AS1/ROR-γt	STAT4-AS1 inhibits Th17 differentiation by binding ROR-γt and blocking its translocation to the nucleus and its binding to the IL-17 promoter.	(112)
**Th2-LCR** **lncRNA cluster**	antisense, sense	lncRNA- DNA		Th2	h, m	MLL/IL-4/IL-5/IL-13	The Th2-LCR lncRNA cluster regulates through histone acetylation, (de-)methylation and chromatin remodeling the Th2 cytokine locus including IFN-γ, IL-4, IL-5 and IL-13.	(56, 113, 114)
**Xloc003810**	–	–	MG-T	Th17, Treg cells	h	Xloc003810/IFN-γ/ TNF-α/IL-1β	Xloc003810 increases thymic Th17/Treg ratio and CD4^+^ PD1^+^ T cells in MG-T, which is accompanied by increased production of IFN-γ, TNF-α and IL-1β.	(115, 116)

BC, breast cancer; CRC, colorectal cancer; HCC, hepatocellular carcinoma; LC, lung cancer; MG-T, myasthenia gravis with thymoma; NHL, non-Hodgkin B-cell lymphoma; h, human; m, mouse.

In CD4^+^ Th1 cells, deep RNA-seq analysis led to the identification of the chromatin-associated linc-MAF-4. This lncRNA is crucial for proper Th1 lineage development, as siRNA-mediated knockdown of linc-MAF-4 induced differentiation into the Th2 lineage. Mechanistically, the 5’ and 3’ region of linc-MAF-4 interacts through chromatin-looping with the promoter of MAF (a Th2-associated transcription factor). The authors go on to show that linc-MAF-4 represents a scaffold to recruit the chromatin modifiers EZH2 and LSD1, which induces repressive H3K27me3 epigenetic marks on the MAF promoter (105). This study beautifully demonstrates the essential Th1 lineage-determining function of linc-MAF-4.

CD4^+^ Th2 cells harbor a lineage-specific Th2-LCR lncRNA cluster, which consists of 4 alternatively spliced transcripts, and regulates the Th2 cytokine locus (IL-4, IL-5 and IL-13) (56, 113, 114). Interestingly, LCR-knockout studies in mice using the Cre-LoxP system revealed that the Th2-LCR lncRNA cluster downregulates IFN-γ (56, 113, 114), a cytokine that is crucial for promoting anti-tumor immune responses (118). Similar to the TH2-LCR lncRNA cluster, the two lncRNAs linc-Ccr2-5’ AS and GATA3-AS1 were shown to be essential for Th2-specific lineage commitment (101, 104). GATA3-AS1 is crucial for the transcription of IL-5, IL-13 and GATA3. Mechanistically, GATA3-AS1 establishes H3K4-methylation and H3K27-acetylation marks at the GATA3 locus during Th2 polarizing conditions by recruiting an MLL methyltransferase via WDR5 (101). GATA3 itself guides the expression of linc-Ccr2-5’ AS, which induces several downstream target genes including the chemokine receptor encoding genes *Ccr2* and *Ccr3*, thereby modulating Th2 cell migration (104). It is intriguing to speculate whether the Th2-LCR lncRNA cluster (linc-Ccr2-5’ AS and GATA3-AS1) is part of the same Th2 regulatory circuit, and whether the TME induces the Th2-LCR lncRNA cluster to facilitate tumor immune evasion by promoting Th2 cell differentiation.

LncRNAs modulating CD4^+^ T cell subsets in a tumor context-dependent manner is a newly emerging field of research. So far, only few lncRNAs have been studied to determine their functional importance in tumor-infiltrating CD4^+^ T cell subsets (**Table 2**). However, lncRNA colorectal neoplasia differentially expressed (CRNDE) is highly expressed in multiple cancer types, and in CRC, lncRNA isoform CRNDE-h is transmitted via tumor-exosomes to induce Th17 differentiation in tumor-infiltrating CD4^+^ T cells. LncRNA CRNDE-h targets the PPXY motif of ROR-γt inhibiting Itch-mediated ubiquitination and degradation of ROR-γt, thereby inducing IL-17 expression (99). This highlights another mechanism of how lncRNAs from tumor cell-derived exosomes are able to modulate the tumor microenvironment to facilitate cancer progression.

Although these findings highlight the relevance of lncRNAs in governing CD4^+^ T cell differentiation into distinct T helper cell lineages, the exact role of these and other lncRNAs in shaping CD4^+^ T cell responses in the TME awaits further investigation.

### 2.3 Regulatory T cells

Treg cell development occurs in the thymus by self-antigen recognition with moderate avidity (thymic Tregs; tTregs) or alternatively, in specialized peripheral tissues (peripheral Tregs; pTregs). In the gut, pTregs develop from CD4^+^ T cells in the presence of TGF-β and retinoic acid (119, 120). The T cell receptor diversity distinguishes tTregs from pTregs. While tTregs primarily engage self-antigens, pTregs can also bind commensal microbiota antigens thereby providing mucosal tolerance (120). Tregs typically express the transcription factor Forkhead box protein 3 (FoxP3), the high affinity IL-2 receptor alpha (CD25) and low levels of IL-7Rα (CD127). However, also FoxP3-independent, IL-10 producing type 1 regulatory cells (Tr1) have been described. Tr1 cells originate extra-thymically, lack FoxP3 but express CD49b and lymphocyte activation gene 3 (LAG-3), produce IL-10 and TGF-β thereby maintaining immune homeostasis and preventing autoimmune diseases (121). Although a recent study discovered that the transcriptional program of pTregs is independent of FoxP3, FoxP3 is still crucial for a specific pTreg function, namely to suppress inflammatory Th17 cells (122). Another FoxP3-dependent subtype are tissue Tregs. They reside in non-lymphoid tissues and originate from a common FoxP3^+^ CD4^+^ precursor in lymphoid organs. Aside from immune surveillance, tissue Tregs regulate non-lymphoid progenitors and function during tissue homeostasis (123).

In cancer, Tregs dampen anti-tumor immune responses by secreting suppressive cytokines (TGF-β and IL-10), consuming excessive amounts of the T cell growth factor IL-2 (thereby impairing effector T cell proliferation), expressing inhibitory receptors (CTLA-4, PD-1 and LAG3) and inducing tolerogenic dendritic cells (98). Generally, intratumoral Tregs are associated with poor prognosis in patients, although differences have been observed between cancer types and studies (36). Treg cell depletion has shown promising results in preclinical cancer models, and anti-CTLA-4 therapy not only increases tumor-specific effector CD4^+^ T cells but also depletes intratumoral CTLA-4^+^ Tregs (98, 124).

Numerous lncRNAs have been shown to be expressed in Tregs or to regulate Treg cell differentiation are Flatr (125), Flicr (126), lnc-SMAD3 (127) and PVT1 (128) (**Figure 1**). Some of these lncRNAs may severe as novel targets for cancer immunotherapy as they have been shown to modulate the complex immunosuppressive TME (**Table 3**).

**Table 3:** LncRNAs regulating Treg cells during homeostasis and cancer.

LncRNA	LncRNA type	Molecular Interaction	Cancer type	T cell subset	Species	Affected Signaling Molecules/Pathway	Function	Ref.
**FENDRR**	intergenic	lncRNA- RNA	HCC	Treg cells	h, m	miR-423- 5p/GADD45B	FENDRR binds to miR-423-5p and upregulates GADD45B. This decreases production of TGF-β and IL-10 by Treg cells, thereby preventing Treg-driven immune evasion in HCC.	(129)
**Flatr**	intronic	–	–	Treg cells	m	FoxP3	Flatr promotes the expression of FoxP3 and enhances the immunosuppressive function of Tregs.	(125)
**Flicr**	intergenic	–	–	Treg cells	h, m	FoxP3	Flicr inhibits the expression of FoxP3 by modifying chromatin accessibility and limits the immunosuppressive function of Tregs.	(126)
**linc- POU3F3**	intergenic	lncRNA- protein	GC	Treg cells	h	TGF-β/SMAD2/3	Linc-POU3F3 promotes Treg cell proliferation by binding TGF-β and inducing TGF-β/SMAD signaling in gastric cancer.	(130)
**lnc-EGFR**	antisense	lncRNA- protein	HCC	Treg cells	h	EGFR, AP-1/ NFAT1/FoxP3 axis	Lnc-EGFR promotes Treg cell differentiation by inhibiting EGFR ubiquitination via c-CBL and subsequent degradation. This amplifies downstream signaling via AP-1 and NFAT1.	(93)
**lnc-INSR**	intergenic	lncRNA- protein	PALL	Treg cells	h	INSR/PI3K/Akt	Lnc-INSR binds to INSR and abnormally activates INSR and PI3K/Akt signaling, thereby enhancing Treg cell differentiation and reducing cytotoxic T cells in PALL.	(131)
**lnc-Smad3**	sense	lncRNA- protein	–	Treg cells	m	TGF-β/Smad3/ HDAC1/ Ash1l	Lnc-Smad3 blocks Smad3 transcription via deacetylation by HDAC1. TGF-β mediated activation of Smad3 represses lnc-Smad3 allowing Ash1l to induce H3K4me3 at Smad3 and FoxP3 promoters to promote iTreg polarization.	(127)
**MIAT**	intergenic	–	HCC	Treg cells, CD8^+^ T cells	h, m	PD-1/CTLA4	LncRNA MIAT correlates with Tregs and exhausted CD8^+^ T cells expressing the co-inhibitory receptors PD-1 and CTLA-4 in HCC.	(75)
**PVT1**	intergenic, sense	lncRNA- protein	–	Treg cells	h, m	PVT1/Myc	In CD4^+^ T cells of Sjörgen’s disease patients, highly expressed lncRNA PVT1 regulates Myc expression, thereby affecting T cell proliferation and effector function.	(128)
**RP11- 323N12.5**	sense	lncRNA- protein	GC	Treg cells	h	c-MYC/YAP1	RP11-323N12.5 induces expression of the transcription factor YAP1, which promotes Treg cell differentiation and GC growth.	(132)
**SNHG1**	intergenic	lncRNA- RNA	BC	Treg cells	h	miR-488/IDO	LncRNA SNHG1 inhibits Treg cell differentiation via regulating miR-488/IDO resulting in tumor immune escape.	(133)

BC, breast cancer; GC, gastric cancer; HCC, hepatocellular carcinoma; PALL, pediatric acute lymphoblastic leukemia; h, human; m, mouse.

For example, lnc-epidermal growth factor receptor (lnc-EGFR) has been described to be highly expressed in Tregs in HCC (93). Mechanistically, lnc-EGFR interacts with EGFR and inhibits its ubiquitination by c-CBL and subsequent degradation while amplifying downstream signaling via AP-1/NFAT1. NFAT1 and AP-1 binding sites were identified in the promotor regions of Foxp3, lnc-EGFR and EGFR. NFAT1 and AP-1 signaling increased the expression of all three genes, indicating a feed forward loop of lnc-EGFR/EGFR/NFAT1/AP-1 in Tregs in HCC (**Figure 3B**) (93). The multi-kinase inhibitor sorafenib and the receptor tyrosine kinase inhibitor lenvatinib are currently being used as first-line treatments against HCC (134) and blocking EGFR has shown to further increase the efficacy of those two drugs (135, 136). Targeting lnc-EGFR and its signaling axis may therefore represent a promising strategy to improve the anti-tumor activities of sorafenib and lenvatinib (93). This model showcases how lnc-EGFR connects an immunosuppressive state to cancer by inducing Treg cell differentiation, suppressing cytotoxic T cells and promoting tumor immune evasion.

Another interesting lncRNA that indirectly modulates the suppressive function of Tregs in HCC is lncRNA fetal-lethal non-coding developmental regulatory RNA (FENDRR) (129). Microarray analysis revealed that lncRNA FENDRR is downregulated in HCC as compared to adjacent normal tissue. Mechanistically, overexpressed lncRNA FENDRR competitively binds miR-423-5p and upregulates “growth arrest and DNA-damage-inducible beta protein” (GADD45B). GADD45B has an anti-tumorigenic function and inversely correlates with Treg cell number. Consequently, its upregulation reduced the Treg cell-associated immunosuppressive cytokines TGF-β and IL-10, and induced apoptosis in tumor cells via Bax and caspase-3 (129). This mechanism nicely demonstrates how downregulation of lncRNA FENDRR in HCC enhances the immunosuppressive ability of Tregs to promote immune escape.

In pediatric acute lymphoblastic leukemia (ALL), lnc-insulin receptor precursor (lnc-INSR) was identified to play a crucial role in promoting suppressive immune cells. High throughput screening revealed an increased expression of lnc-INSR in Tregs in the bone marrow of pediatric ALL patients. A combination of FISH and RIP assays identified the cytoplasmic domain of INSR as the target of lnc-INSR. Consequently, INSR and its downstream signaling pathway PI3K/Akt is abnormally activated causing immune suppression and tumor growth via enhanced Treg cell differentiation and a reduction in cytotoxic T cells (131).

Additionally, lncRNA MIAT, SNHG1, lnc-POU3F3 and RP11-323N12.5 have been associated with Tregs in cancer. LncRNAs linc-POU3F3 and RP11-323N12.5 are both associated with gastric cancer (GC) (130, 132). Linc-POU3F3 was upregulated in peripheral blood CD4^+^ CD25^+^ FoxP3^+^ Tregs of GC patients and could recruit TGF-β to induce its downstream signaling pathway which triggered Treg proliferation (130). RP11-323N12.5 was highly expressed in human GC cells. Functionally, RP11-323N12.5 stimulates the expression of the transcription factor YAP1. YAP1 itself promotes lncRNA RP11-323N12.5 expression as part of a positive feedback loop. RP11-323N12.5 was also detected in tumor-infiltrating lymphocytes such as Tregs, and enhanced Treg cell differentiation thereby shifting the balance towards an immunosuppressive TME (132). Another lncRNA, lncRNA SNHG1 was identified in breast cancer-infiltrating CD4^+^ T cells. LncRNA SNHG1 bound miR-448, which decreased IDO expression and increased FoxP3 and IL-10 levels in Tregs. SiRNA-mediated knockdown of SNHG1 decreased tumor progression indicating that SNHG1 promotes tumor immune escape by stimulating Treg cell differentiation (133). In HCC, lncRNA MIAT expression was upregulated in tumor cells, FoxP3^+^ Tregs, PD-1^+^ CD8^+^ T cells and GZMK^+^ CD8^+^ T cells (75). MIAT expression correlated with the responsiveness to anticancer drugs, such as sorafenib, and with the expression of PD-L1. However, additional functional analyses are needed to reveal the molecular mechanism and the role of MIAT in tumor cells and different immune cell types. Additionally, two lncRNAs, Flatr and Flicr both modulate FoxP3 expression on Tregs. While Flatr is part of the upstream signaling cascade triggering FoxP3 expression (125), Flicr functions as a negative regulator modulating FoxP3 expression under IL-2 deficient conditions. Thereby, Flicr limits Treg activity promoting autoimmunity (126). Consequently, this mechanism could be exploited for Treg-cell therapy to reduce immunosuppression by Tregs in cancer.

Another lncRNA that affects the Treg/CD8^+^ T cell balance in the TME is lnc-Sox5. In colorectal cancer (CRC), lnc-Sox5 induces indoleamine 2,3-dioxygenase 1 (IDO1) expression, which is known to promote Treg cell differentiation. Conversely, the inhibition of lnc-Sox5 limits Treg cell differentiation thereby reducing tumorigenesis (88). Similarly, LINC00301 induces an immunosuppressive tumor microenvironment by modulating the CD8^+^ T cell/Tregs ratio in non-small cell lung cancer (NSCLC) (137). Interestingly, this lncRNA regulates Tregs by targeting hypoxia-inducible factor 1α (HIF-1α) in two different ways: nuclear LINC00301 binds to EZH2, which downregulates the EAF2 promoter and increases HIF-1α expression, whereas cytoplasmic LINC00301 targets HIF-1α via miR-1276.

Altogether, these studies demonstrate the relevance of lncRNAs in Treg cell differentiation and in establishing an immunosuppressive TME to promote tumor growth and tumor immune evasion. Several lncRNAs have been linked to the complex interplay between Tregs and the TME, and may therefore represent interesting therapeutic targets.

### 2.4 γδ T cells and NKT cells

In humans, two major subsets of γδ T cells have been identified. Vδ1 T cells are found in the thymus and peripheral tissues, and recognize stress-related antigens. And Vδ2 T cells are found in the circulation and mostly recognize phospho-antigens. One feature that distinguishes γδ T cells from conventional αβ T cells is their ability to identify tumor cells regardless of human leukocyte antigen (HLA) restriction. In a number of cancer entities, a considerable part of the tumor-infiltrating lymphocyte population consists of γδ T cells (138). The abundance of γδ T cells, in particular Vδ1 T cells, has been associated with favorable prognosis in some cancers, such as non-small cell lung cancer (NSCLC) (139), triple-negative breast cancer (TNBC) (140) and ovarian cancer (141). However, tumor-infiltrating γδ T cells can also differentiate into pro-tumorigenic IL-17^+^ γδ T cells or immunosuppressive γδ T regulatory cells (138) thereby being associated with poor patient prognosis. A recent study indicates that TCR-Vγδ usage distinguishes pro-tumor from anti-tumor intestinal γδ T cell subsets (142). Due to their better functional characterization and their frequent abundance in numerous cancer types, γδ T cells are recognized as a promising cellular target for cancer immunotherapy (143), and lncRNAs could act as molecular targets to specifically modulate γδ T cell function in the TME (**Table 1**).

In a study by Ni et al. (82), the exosomal lncRNA SNHG16 was identified in γδ T cells of breast cancer (BC) patients. Taking advantage of co-culture experiments, microarray and mechanistic analyses, the authors demonstrated that breast tumor cell-derived exosomes can transfer lncRNA SNHG16 to γδ T cells. The SNHG16/miR-16-5p/TGF-β1/SMAD5 pathway increased CD73 expression in γδ T cells, thereby inducing an immunosuppressive CD73^+^ Vδ1 T cell subpopulation (82). This work nicely exemplifies exosomal transmission of a lncRNA from a tumor cell to γδ T cells, which promotes a regulatory CD73^+^ Vδ1 T cell subset and thus facilitates tumor immune evasion.

LncRNA TANCR is another recently identified human γδ T cell-specific lncRNA. Deep RNA sequencing, led to the discovery of TANCR in isopentenyl pyrophosphate (IPP)-activated γδ T cells generated from human PBMCs. This study indicates that TANCR regulates γδ T cell activation and modulates tumor necrosis factor-related apoptosis-inducing ligand (TRAIL) expression (83). Thereby, TANCR may induce apoptosis of target cells, which could be utilized for therapeutic purposes. However, additional functional studies will be required to identify the molecular mechanism of TANCR and its regulation in the TME.

NKT cells are another T cell subpopulation that participates in the immune surveillance of tumors. In cancer patients, exogenous NKT cell activation triggers a considerable immune response and NKT cell infiltration into tumors correlates with a favorable prognosis in a variety of cancer types (144). Currently, only one lncRNA has been identified that modulates NKT cell function in cancer. In a recent study, the intergenic lncRNA LINC00240 was found to be increased in cervical cancer. Mechanistic analyses demonstrated that LIN00240 promotes cancer progression by sponging miR-124-3p and activating STAT3, which caused NKT cell tolerance (70).

## 3 The impact of tumor cell-expressed lncRNAs on T cell responses

LncRNAs have been shown to be expressed in all major cancer types, contribute to the hallmarks of cancer, and can act as oncogenes (“onco-lncRNAs”, such as HOTAIR, NEAT1 and MALAT1) or tumor suppressors (e.g. GAS5, MEG3, NBAT and LINC-PINT) (67, 145–147). Numerous lncRNAs have been shown to regulate tumorigenesis, epithelial-mesenchymal transition and metastasis, and some of those lncRNAs may prove useful for risk stratification of cancer patients or may be included in the molecular classification of cancer subtypes (63, 65). In this section, we will highlight tumor cell-expressed lncRNAs that drive immune escape by modulating T cell infiltration or T cell dysfunction/exhaustion (**Table 4**) and that may therefore represent promising targets for therapeutic intervention.

**Table 4:** Tumor cell-expressed lncRNAs that modulate TIL populations.

LncRNA	LncRNA type	Molecular Interaction	Cancer type	T cell subset	Species	Affected Signaling Molecules/Pathway	Function	Ref.
**SNHG20**	intergenic	–	ESCC	T cells	h	ATM/JAK/PD-L1	LncRNA SNHG20 regulates p-ATM, pJAK1/2 and PD-L1 expression and drives metastasis and ESCC progression.	(148)
**Malat1**	intergenic	lncRNA- RNA	DLBCL	CD8^+^ T cells	h, m	miR-195/PD-L1	LncRNA Malat1 induces tumor growth and immune evasion in DLBCL by sponging miR-195 to regulate PD-L1 expression.	(149)
**INCR1**	antisense	lncRNA- protein	GBM, BC, NSCLC, melanoma	CD8^+^ T cells	h	HNRNPH1/PD- L1/JAK2/IFN-γ signaling	IFN-γ-stimulated lncRNA INCR1 binds HNRNPH1 to promote PD-L1 and JAK2 expression. Silencing *INCR1* sensitizes tumor cells to T cell-mediated killing.	(89)
**LIMIT**	antisense	lncRNA- DNA	Melanoma	CD8^+^ T cells	h, m	GBP/HSF1/MHC	IFN-γ-induced LIMIT stimulates the GBP cluster, activates HSF1 and thereby increases MHC-I but not PD-L1. LIMIT correlated with MHC-I and ICI therapy response in patients.	(150)
**EPIC1**	intergenic	lncRNA- protein	32 cancer types	CD8^+^ T cells	h	EZH2/IFNγ-JAK- STAT1 signaling	EPIC1 blocks IFNγ-JAK-STAT1 signaling via epigenetic silencing of IFNGR1, which decreases MHC-I expression and CTL infiltration into tumors.	(92)
**LINK-A**	Intergenic	lncRNA- protein	TNBC	CD8^+^ T cells	h, m	LINK-A/ PKA/TRIM71	LINK-A (LINC01139) acts as an oncogene by downregulating cancer cell antigen presentation via suppression of PKA-mediated phosphorylation of the E3 ubiquitin ligase TRIM71.	(151)
**NEAT1**	intergenic	lncRNA- protein	LC	CD8^+^ T cells	h, m	DNMT1/cGAS- STING/P53	LncRNA NEAT1 promotes lung cancer growth and decreases tumor-infiltrating T cells by interacting with DNMT1 thereby inhibiting the expression of cGAS/STING and P53.	(152)
**SOX2-OT**	intergenic	lncRNA- RNA	NSCLC	CD8^+^ T cells	h	miR-30d-5p/ PDK1/mTOR/PD-L1	SOX2-OT upregulates PDK1 expression by sponging miR- 30d-5p and drives PD-L1 through mTOR signaling.	(153)
**KCNQ1OT1**	antisense	–	CRC	CD8^+^ T cells	h	CD155	KCNQ1OT1 is a prognostic biomarker, modulates CD155 expression, thereby induces CD8^+^ T cell exhaustion in CRC.	(154)
**LINC00473**	intergenic	lncRNA- RNA	PC	CD8^+^ T cells	h	miR-195-5p/PD-L1	LINC00473 drives tumorigenesis by sponging miR195-5p, increases PD-L1 thus inhibiting CD8^+^ T cell activation.	(155)
**SNHG14**	intergenic	lncRNA- RNA	DLBCL	CD8^+^ T cells	h, m	miR-5590-3p/ ZEB1/PD-L1	SNHG14 sponges miR-5590-3p, upregulates ZEB1 and promotes SNHG14 and PD-L1 expression, thereby driving DLBCL progression and immune escape.	(156)
**HCG18**	-	lncRNA- RNA	CRC	CD8^+^ T cells	m	HCG18/miR-20b-5p/ PD-L1	LncRNA HCG18 expressed in CRC inhibits CD8^+^ T cells and induces resistance to cetuximab. Functionally, HCG18 enhances PD-L1 expression by sponging miR20b-5p.	(157)
**SNHG1**	intergenic	lncRNA- RNA	RCC	CD8^+^ T cells	h	SNHG1/miR-129-3p/ STAT3/PD-L1	SNHG1 targets STAT3 / PD-L1 by binding miR-129-3p. This promotes tumor immune evasion by inhibiting CD8^+^ T cells.	(158)
**lnc-Sox5**	-	lncRNA- protein	CRC	CD8^+^ T cells, Treg cells	h	IDO1	Lnc-Sox5 suppresses infiltration and cytotoxicity of CD8^+^ T cells by promoting Treg cells via IDO1.	(88)
**LINC00301**	intergenic	lncRNA- protein lncRNA- RNA	NSCLC	CD8^+^ T cells, Treg cells	h, m	FOXC1/LINC00301/ EZH2 or miR-1276/ HIF-1α	LINC00301 modulates the TME by increasing the Treg/CD8^+^ T cell ratio. It either targets nuclear EZH2 or cytoplasmic miR-1276 to regulate HIF-1α.	(137)
**NNT-AS1**	antisense	-	HCC	CD4^+^ T cells	h	TGF-β signaling	NNT-AS1 is a potential biomarker in HCC. It activates the TGF-β pathway and decreases tumor-infiltrating CD4^+^ T cells.	(159)
**HOXA-AS2**	antisense	lncRNA- RNA	GBM	Treg cells	h	HOX-AS2/miR-302a/ KDM2a/JAG1	LncRNA HOXA-AS2 targets miR-302a, which stimulates the KDM2A/JAG1 pathway to promote Treg cell proliferation.	(160)
**circRNA- 002178**	circular	lncRNA- RNA	LUAD	CD8^+^ T cells	h	PD-L1/PD-1	CircRNA-002178 enhances PD-L1 expression via sponging miR-34 in cancer cells to induce T cell exhaustion. CircRNA- 002178 can also be transmitted via exosomes to induce PD-1 expression in CD8^+^ T cells.	(161)
**circIGF2BP3**	circular	lncRNA- RNA	NSCLC	CD8^+^ T cells	h	miR328-3p/miR3173- 5p/PKP3/FXR1/ OUTB1/PD-L1	CircIGF2BP3 blocks CD8^+^ T cell infiltration by sponging miR328-3p and miR3173-5, which activates the PKP3/FXR1/ OUTB1 axis to promote PD-L1 expression in NSCLC.	(162)
**circUSP7**	circular	lncRNA- RNA	NSCLC	CD8^+^ T cells	h	miR-934/SHP2	The exosomal circUSP7 promotes CD8^+^ T cell dysfunction and resistance to anti-PD1 therapy by sponging miR-934 and inducing SH2 expression.	(163)

BC, breast cancer; CRC, colorectal cancer; DLBCL, diffuse large B-cell lymphoma; ESCC, esophageal squamous cell carcinoma; GBM, glioblastoma; HCC, hepatocellular carcinoma; LC, lung cancer; LUAD, lung adenocarcinoma; NSCLC, non-small cell lung cancer; PC, pancreatic cancer; RCC, renal cell carcinoma; TNBC, triple-negative breast cancer; h, human; m, mouse.

Using whole-transcriptome analysis, lncRNA IFN-stimulated non-coding RNA 1 (INCR1) was identified as a crucial modulator of IFN-γ signaling in multiple tumors (89). INCR1 confers its function by post-transcriptionally regulating PD-L1 and JAK2 activity. Mechanistically, the nuclear riboprotein HNRNPH1 binds and downregulates PD-L1 and JAK2. However, INCR1 can competitively bind HNRNPH1, which enables PD-L1 and JAK2 expression. Knockdown of INCR1 repressed IFN-γ-stimulated genes (ISGs) and increased T cell-mediated killing of tumor cells. Intriguingly, *in vivo* silencing of INCR1 improved CAR T cell therapy responses (89).

While INCR1 serves as an inhibitory target to promote IFN-γ response and T cell activity, another cancer immunogenic lncRNA, lncRNA inducing MHC-I and immunogenicity of tumor (LIMIT), has been identified recently (150). The IFN-γ-induced lncRNA LIMIT stimulates the guanylate-binding protein (GBP) gene cluster in *cis*. Consequently, the HSP90 and heat shock factor-1 (HSF1) complex dissociate, activating HSF1 and increasing MHC-I expression but not PD-L1. Li et al. also performed RNA-guided CRISPR activation of LIMIT, which boosted GBPs and MHC-I, and potentiated tumor immunogenicity and anti-PD-L1 antibody therapy. Remarkably, the authors performed CRISPR activation of LIMIT in a B16 melanoma model, which characteristically lacks responsiveness to PD-L1 blockade, and demonstrated resensitization of the tumor to anti-PD-L1 therapy. Since the loss of MHC-I and IFN-γ is commonly observed in cancer (150), lncRNA LIMIT provides a novel therapeutic opportunity to increase MHC I on tumors and to resensitize cancers to anti-PD-L1 therapy.

Another lncRNA that may act as a target for immunotherapy is lncRNA EPIC1 (**Figure 3C**). For the identification of lncRNA EPIC1, Guo et al. employed an interesting new approach to dissect the landscape of interactions between lncRNAs and tumor immunity (92). Based on bulk tumor RNA-seq data collected in The Cancer Genome Atlas (TCGA) database comprising 32 cancer types and more than 9000 tumor samples, they performed an integrative analysis of lncRNA expression and immunogenomics profiles of the TME. This led to the development of a lncRNA-based immune response (LIMER) score which, predicts lymphocyte infiltration of tumors and patient prognosis. Tumor tissue-specific lncRNA EPIC1 correlated with lower infiltration and activation of CD8^+^ T cells as well as decreased tumor antigen presentation in multiple cancers. Mechanistically, lncRNA EPIC1 interacts with EZH2, a histone methyltransferase that epigenetically silences IFNGR1 and consequently IFN-γ signaling (92). Thereby, the authors propose a novel and distinct therapeutic target for immunotherapy across multiple cancer types.

The lncRNA LINK-A (*LINC01139*) acts as an oncogene by downregulating cancer cell antigen presentation as well as the tumor suppressors p53 and Rb (151). LINK-A is upregulated in multiple cancer types, including triple-negative breast cancer, and high LINK-A expression correlates with low CD8^+^ T cell abundance in basal-like breast cancer. Mechanistically, LINK-A binds to phosphatidylinositol-(3,4,5)-triphosphate, suppressing protein kinase A-mediated phosphorylation of the E3 ubiquitin ligase TRIM71. As a consequence, LINK-A enhances K48-polyubiquitination-mediated degradation of the antigen peptide-loading complex as well as p53 and Rb. Using a mouse model, treatment with LINK-A locked nucleic acids (LNAs) sensitized breast tumors to immune checkpoint blockade therapy (anti-PD-1 + anti-CTLA-4) thus providing a basis for a rational combination immunotherapy regimen (151).

Apart from tumor-expressed lncRNAs that impair effector CD8^+^ T cells (88, 89, 92, 137, 150), several lncRNAs have been described that induce an immunosuppressive TME by altering tumor-infiltrating CD4^+^ T cells (137, 159), Tregs (88, 129, 137, 159, 160), γδ T cells (82, 83) and NKT cells (70). For example, lncRNA NNT-AS1 impairs TGF-β signaling and reduces CD4^+^ T cell tumor infiltration, and thereby promotes HCC progression and metastasis (159, 164). Another lncRNA, HOXA-AS2 has been shown to contribute to Treg cell proliferation and immune tolerance in glioma through the miR-302a/KDM2A/JAG1 axis (160).

The above-mentioned studies demonstrate how tumor cell-expressed lncRNAs promote an immunosuppressive TME by modulating MHC I expression or several signaling pathways, such as IFN-γ and TGF-β. As IFN-γ signaling is an integral part of anti-tumor immunity, these lncRNAs, in particular lncRNA EPIC1 due to its pan-cancer applicability, may represent a promising target for therapeutic intervention.

## 4 Challenges for lncRNA-based therapeutics

Several siRNAs that target mRNA transcripts are currently in clinical use or development (8, 165). Although no lncRNA-based therapeutics have entered the clinic yet, there are several strategies to inhibit lncRNAs, depending on their mode of action. Second-generation antisense oligonucleotides (ASOs), which target pre-mRNA splicing, as well as third-generation LNA oligonucleotides represent promising therapeutics to inhibit lncRNAs. In general, small-molecule inhibitors have two modes of action to block lncRNA function: Interaction element blockers (IEBs), that block RNA/DNA/protein-docking sites on lncRNAs, and structural element lockers (SELs), which affect the conformation of a lncRNA thereby inhibiting functional interactions with interactor molecules (8). One example of an IEB is NP-C86 that stabilizes lncRNA GAS5 by preventing its interaction with UPF1, which normally mediates nonsense-mediated decay of GAS5 (166). NP-C86 may thus prevent the decay of the tumor suppressor GAS5 and inhibit Th17 cell differentiation (**Figure 1**) (107). There are also SELs being developed for MALAT1, which aim to destabilize and down-regulate MALAT1, albeit their efficacy and specificity still need to be evaluated (167). MALAT1 also contributes to enzalutamide resistance in castration-resistant prostate cancer, and a MALAT1 siRNA reduced growth of enzalutamide-resistant tumor xenografts (168). Despite the frequently reported pro-tumorigenic and metastasis-promoting role of MALAT1 (169), a study by Kim et al. found that MALAT1 can also act as a tumor suppressor in a mouse model of breast cancer (170). Since MALAT1 is expressed in various cell types, and affects the function of CD8^+^ and CD4^+^ T cells (74, 171), it remains to be seen whether SELs or siRNAs that target MALAT1 are effective in inhibiting the growth of different tumor types. In summary, some progress has been made to advance therapeutics to target noncoding RNAs. However, there are still some important challenges ahead, such as improving specificity and delivery as well as reducing immunogenicity and toxicity of lncRNA-based therapeutics. A detailed description of RNA therapeutics that are in clinical development or that have been approved as well as the challenges that remain for the development of RNA-based therapeutics has been summarized in an excellent perspectives article by Winkle and colleagues (8).

## 5 Conclusions

LncRNAs have been recognized for their unique ability to regulate gene expression, nuclear architecture and cellular function. LncRNAs have been identified as critical regulators of T cell-mediated immunity during homeostasis, infection and cancer, highlighting the use of lncRNAs as potential biomarkers and/or more precise therapeutic targets. Advances in CRISPR-Cas genome editing have enabled high throughput functional screens and accelerated the generation of knockout and transgene mouse models to properly address the functional significance of lncRNAs (145, 172). This will facilitate uncovering the complex role of lncRNAs in shaping an immunosuppressive tumor microenvironment or in contributing to metastasis and immunotherapy resistance. Combining different technologies will be of great importance in order to investigate whether a lncRNA locus has multiple functional modalities, such as mediating its function through the RNA molecule itself, DNA elements encoded in the locus or via transcription (173). A lncRNA may have many molecular interactions and multiple context-specific and/or cell-type specific functions. Additionally, large lncRNA loci may overlap with other regulatory elements and may therefore generate multiple different RNAs or encode multiple different DNA elements. PVT1 and MALAT1 are examples of two lncRNA loci that have multiple and/or opposing effects (63). In conclusion, lncRNAs have been shown to be key regulators of T cell-mediated responses, and the recent technological advances will prove useful to provide definitive evidence for the functional relevance of lncRNAs in different cell types and context-specific environments. However, major challenges remain before transferring research results to the clinic, such as improving specificity and delivery as well as reducing immunogenicity and toxicity of lncRNA-targeting therapeutics.

## Author contributions

Conceptualization: DH-B. Writing - original draft: JE. Writing - review and editing: DH-B. Funding acquisition: DH-B. All authors read and approved the submitted version.
